# Duhuo Jisheng decoction alleviates neuroinflammation and neuropathic pain by suppressing microglial *M*1 polarization: a network pharmacology research

**DOI:** 10.1186/s13018-023-04121-9

**Published:** 2023-08-28

**Authors:** Chengcan Gao, Yulong Zhao, Tao Yang, Xu Gao, Chunyang Meng

**Affiliations:** 1 Department of Surgery, Jining No. 1 People’s Hospital, Jining, 272000 Shandong China; 2 Shandong First Medical University, Jinan, 250117 Shandong Province China; 3 Department of Spine Surgery, Affiliated Hospital of Jining Medical University, 89 Guhuai Road, Jining, 272000 Shandong Province China; 4 Department of Orthopaedic Surgery, Qingdao University, Qingdao City, 266071 China

**Keywords:** Duhuo Jisheng decoction, Network pharmacology, Neuropathic pain, Inflammation, Microglial, IL-17 signaling pathway

## Abstract

**Background:**

Neuropathic pain (NP) is the most prevalent form of chronic pain resulting from nerve damage or injury. Despite the widespread use of Duhuo Jisheng decoction (DHJSD) in traditional Chinese medicine (TCM) to treat chronic pain, the mechanism underlying its analgesic action remains unclear.

**Methods:**

Using network pharmacology, we obtained DHJSD and NP-related target information from public databases to construct protein–protein interactions (PPI) and compound-target networks based on common target genes. These networks were further analyzed using gene ontology (GO) and Kyoto encyclopedia of genes and genomes (KEGG). The interaction between molecules was verified through molecular docking using AutoDock Tools software. Additionally, we treated a chronic constriction injury (CCI) rat model with DHJSD and determined the mechanical withdrawal threshold (MWT). We used an enzyme-linked immunosorbent assay kit to determine the levels of inflammatory cytokines. Furthermore, qRT-PCR was employed to analyze ACHE, NOS2, MAPK3, PTGS2, AKT1, and PPARG mRNA expression, and immunofluorescence was used to evaluate changes in microglia.

**Results:**

Our screening of compounds and targets identified 252 potential targets of DHJSD associated with NP. PPI analysis, along with GO and KEGG analyses, revealed that the potential mechanism of DHJSD in NP treatment may be related to inflammatory reactions, the IL-17 signaling pathway, MAP kinase activity, and endocrine activity. Based on molecular docking, the core target showed significant affinity for DHJSD's active components. Moreover, DHJSD treatment repaired the CCI-induced inflammatory reaction in the spinal cord while regulating the expression of ACHE, NOS2, MAPK3, PTGS2, AKT1, and PPARG mRNA. Immunofluorescence results indicated that the active components of DHJSD may regulate microglial *M*1 polarization to improve neuroinflammation, PPARG may have been involved in the process.

**Conclusion:**

The multi-component, multi-target, and multi-pathway actions of DHJSD provide new insights into its therapeutic mechanism in NP.

**Supplementary Information:**

The online version contains supplementary material available at 10.1186/s13018-023-04121-9.

## Introduction

Chronic pain is a common health condition that affects 6.2% of the general population worldwide. Neuropathic pain (NP) is the most common form of chronic pain caused by nerve damage or injury [1]. Various clinical drugs are prescribed for the treatment of NP, such as antidepressants, antiepileptics, opioids, and local anesthetics [2]; however, these drugs have limited therapeutic effects and intense side effects. Therefore, new analgesic drugs with relatively high efficiency and mild or no side effects are needed.

Traditional Chinese Medicine (TCM) monomers and compounds have been shown to be effective in treating NP [3]. For instance, Wu-tou decoction causes analgesia by inhibiting the activation of hippocampal microglia, alleviating the imbalance between hippocampal glutamatergic and gamma-aminobutyric acid (GABA)ergic neurons [4], and alleviates mechanical pain by modulating the inflammatory response through the TREM2-autophagic axis in the chronic constriction injury (CCI) model [5]. Moreover curcumin and puerarin can alleviate peripheral neuropathic pain by inhibiting oxidative stress-mediated NF-κB activation, thereby reducing inflammation [6, 7].

Angelicae Pubescentis Radix (APR), known as Duhuo Jisheng Decoction (DHJSD), is a medicinal herb widely used to treat sciatica, rheumatoid arthritis, and osteoporosis. APR Extracts and their constituent compounds have potent analgesic and anti-inflammatory effects [8]. For instance, inflammatory factors and TRPV1 mediate coumarins in damaged dorsal root ganglion neurons, decreasing pro-inflammatory cytokines (TNF-a, IL-1β, and IL-6) levels as well as TRPV1 and pERK expressions [9]. DHJSD includes numerous other components with unknown actions that need to be studied to expand its use in Traditional Chinese Medicine (TCM) (Additional file 1).

Network pharmacology uses biological networks as targets to analyze the links among drugs, targets, and diseases. A comprehensive and systematic study using network pharmacology is consistent with its holistic nature; thus, it is an important tool for screening active components and elucidating their underlying mechanisms of action. Molecular docking was originally used to detect recognition mechanisms between small and large molecules. In recent years, analytical docking has been applied to drug discovery [10], where it is used to identify innovative treatment compounds of interest, predict ligand–target interactions, or describe structure–activity associations at the molecular level. Advances in technology and dramatic increases in chemical and biological information have led to a surge in the use of analytical docking in the medical field, particularly for developing novel drugs [11, 12]. Herein, analytical docking was used to analyze the core target genes, and chemo-small analyses of a network pharmacological screen were used to identify chemo-small molecules that play a role in DHJSD (Additional file 2).

**Fig. 1:**
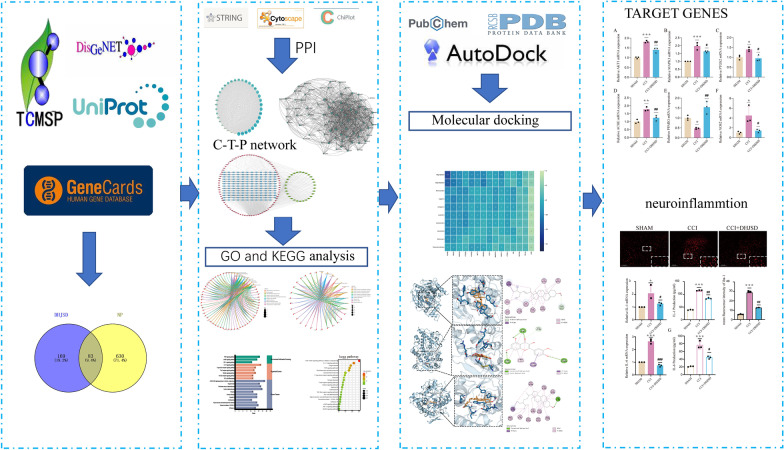
Flowchart of the study

In this study, we aimed to identify and analyze the active components, target genes, and related signaling pathways of DHJSD and validate the results in a CCI rat model to elucidate the mechanism of action of DHJSD on NP (Fig. 1). Our data may help develop new analgesic drugs for the effective treatment of NP.

## Materials and methods

### Identification of active compounds and target genes

By accessing the Traditional Chinese Medicine Systems Pharmacology Database and Analysis Platform (TCMSP), the chemical components of DHJSD were identified, and their possible targets were predicted. The criteria for identifying active components were oral bioavailability (OB) ≥ 30% and drug-likeness (DL) ≥ 0.18 in the following processes: absorption, metabolism, distribution, and excretion. The potential target genes were identified using GeneCards (http://www.genecards.org/) and DisGeNET (https://www.disgenet.org/home/). Target protein names and their corresponding genes were identified and corrected using UniProt (http://www.uniprot.org). Common target genes from the two databases were analyzed using STRING 11 (https://string-db.org) to determine potential protein interactions with protein–protein interaction (PPI) scores > 0.4, indicating significance. Cytoscape 3.8.2 (http://www.cytoscape.org) was used to visualize the PPI network and Wiki pathways. Gene Ontology (GO) and Kyoto Encyclopedia of Genes and Genomes (KEGG) pathway enrichment analyses were conducted to evaluate target genes at the biological functional level, and key signaling pathways were selected based on enrichment scores. Active compounds, target genes, and prominent signaling pathways were used to construct a compound-target pathway network using Cytoscape 3.8.2. Visualization was performed using https://www.bioinformatics.com.cn and https://www.chiplot.online/

### Molecular docking

The affinity of the active components for the crystal structures of the target proteins was verified using PubChem (https://pubchem.ncbi.nlm.nih.gov/) and the Protein Data Bank (PDB, http://www.rcsb.org/pdb). Receptor proteins were retrieved from the PDB. By utilizing PyMOL 2.3.4 (https://pymol.org/2/) and AutoDock Tools (http://autodock.scripps.edu/resources/adt), receptors were modified by running these processes: dehydration, de-liganding, hydrogenation, and equilibrium charge. AutoDock Vina 1.1.2 (http://vina.scripps.edu) was used to dock the receptors to small-molecule ligands.

### Preparation of DHJSD

The 15 herbs used in DHJSD were purchased from Beijing Tong Ren Tang (Beijing, China), ground into a powder, and mixed in relative proportions, as described in the Chinese Pharmacopoeia (http://wp.chp.org.cn/front/chpint/en/, Table 1). Stock solutions were prepared by DHJSD powder decoction in water to a 2 g/mL concentration and kept at -20 °C until further use.

**Table 1:** The 15 herbs of Duhuo Jisheng decoction

Name	Proportionality
*Duhuo Jisheng decoction*	
Angelica pubescens	3
Saposhnikovia divaricata	2
Ligusticum chuanxiong	2
Achyranthes bidentata	2
Loranthus parasiticus	2
Gentiana macrophylla	2
Eucommia ulmoides	2
Angelica sinensis	2
Poriacocos	2
Codonopsis pilosula	2
Radix rehmanniae preparata	2
Radix paeoniae alba	2
Asarum sieboldii	2
Glycyrrhiza uralensis	2
Cinnamomum cassia	2

### Chronic constriction injury (CCI) rat models and treatment

Forty male Sprague Dawley rats (200–250 g) were procured from Jinan Ponyue Experimental Animal Breeding (Jinan, China) and housed under a 12-h diurnal cycle. The rats were provided with water and food ad libitum. The CCI rat models were designed using previously described procedures [13]. Briefly, 1% sodium pentobarbital (40 mg/kg body weight) was administered intraperitoneally to anesthetize rats, followed by loose tying of three knots around the exposed left sciatic nerve at the mid-thigh by 4–0 chromic catgut ligation at 1 mm intervals, while the exposed left sciatic nerve in the sham group was not ligated. The rats were randomized into the sham (control), CCI, and CCI + DHJSD groups. The CCI + DHJSD group was treated daily with DHJSD at 10.3 g/kg [14] body weight, starting on day 1 postoperatively. The CCI group received an equal concentration of saline for seven days, starting on postoperative day 1. This study was approved by the Animal Ethics Committee of JiNing Medical College (2021B099).

### Behavioral analysis

The rats were then transferred to a vitreous chamber with a wired-mesh floor and allowed to acclimate for 30 min. Mechanical allodynia was assessed using the mechanical withdrawal threshold (MWT) at 0, 1, 4, and 7-day postoperation between 8:00 and 17:00. Measurement of 50% was done with von Frey filaments (0.6 g, 1.0 g, 1.4 g, 2.0 g, 4.0 g, 6.0 g, 8.0 g, 10.0 g, and 15.0 g) (North Coast Medical Company, USA) using an up-down approach [15, 16]^.^

### Reverse transcription quantitative PCR (RT-qPCR)

Following the behavioral testing seven days post-operation, rats (*n* = 3/group) were subjected to anesthetize intraperitoneally by administration of 1% sodium pentobarbital (40 mg/kg body weight); the ipsilateral L4-6 spinal cord tissue was rapidly excised, succeeded by using cold phosphate-buffered saline (PBS) for wash and allowed to snap-freeze in liquid nitrogen and − 80 °C storage to be used in further analyses. RNA from the ipsilateral spinal cord was extracted using TRIzol reagent (Ambion, Austin, TX, USA) following the manufacturer’s protocols, and its quantity and purity were assessed at 260/280 nm using a NanoDrop 2000 (Thermo Fisher Scientific, Waltham, MA, USA). The reverse transcription kit (Biosharp, Anhui, China) was used for synthesizing cDNA, conducting RT-qPCR on a Bio-Rad iQ5 real-time system (Bio-Rad, Hercules, CA, USA) using SYBR mixture (Cwbio, Beijing, China) following the protocols, setting the thermal conditions to 25 °C, 55 °C, and 85 °C for 10, 30, and 5 min, respectively, using GAPDH as the reference gene. Primers (Table 2) were procured from Sangon Biotech (Shanghai, China).

**Table 2:** The primer sequence for RT-PCR

Name	Primer	Sequence
RAT	Forward	GAGTCCACTGGCGTCTTCA
GAPDH	Reverse	GGTCATGAGTCCTTCCACGA
RAT	Forward	TTATGCCACCAGAGCCCAAG
ACHE	Reverse	GGAGAAGTAGGCCTGGGGTA
RAT	Forward	AGTCAACTACAAGCCCCACG
NOS2	Reverse	GCAGCTTGTCCAGGGATTCT
RAT	Forward	GGGCCAAGCTTTTTCCCAAA
MAPK3	Reverse	AGCCACTGGTTCATCTGTCG
RAT	Forward	CTCAGCCATGCAGCAAATCC
PTGS2	Reverse	GGGTGGGCTTCAGCAGTAAT
RAT	Forward	AGCTCTGTGGACCTCTCTGT
PPARG	Reverse	GTCAGCTCTTGTGAACGGGA
RAT	Forward	GGAGAAGTTAGAGTCACAGAA
IL-6	Reverse	TGCCGAGTAGACCTCATAG
RAT	Forward	CTCGTGGGATGATGACGACC
IL-1	Reverse	AGGCCACAGGGATTTTGTCG

### Enzyme-linked immunosorbent assay (ELISA)

ELISA was conducted using the L4–6 spinal cords ipsilateral to the nerve injury site. To assess the supernatant collected by homogenizing the spinal cord tissues in cold phosphate buffer, followed by sonication and 15 min of centrifugation at 10,000 rpm, the corresponding rat ELISA kits (Enzyme-linked Biotechnology, China) were used.

### Immunofluorescence

The anesthetized rats (described above) were perfused with 200 mL of cold PBS. The ipsilateral L4-6 spinal cord tissue was rapidly excised, fixed in 4% PFA, embedded in paraffin, and sectioned. Paraffin sections were then sequentially dewaxed in xylene, anhydrous ethanol, 85% alcohol, and 75% alcohol, and placed in antigen retrieval buffer (100 × EDTA, pH 8.0). PBS (pH 7.4) was then used to wash the sections three times (5 min each), followed by 30 min of permeabilization with 0.3% Triton X-100 and a second wash with PBS, after which the sections were blocked for 1 h using 3% BSA. For co-staining, PPARG (1:100; Abcam), Iba-1 (1:500, Abcam, Cambridge, United Kingdom) and CD86 (1:100, Abcam) were co-incubated at 4 °C overnight, followed by 3 times of washing (5 min each) and 2 h of incubation in the dark at 20 °C in the presence of Alexa Fluor-488 or -594 goat anti-rabbit (1:500, Abcam), and counterstained with DAPI at 20 °C for 10 min in the dark. The analysis was performed using a 3DHISTECH slide scan system (CaseViewer; 3DHISTECH Ltd., Hungary).

### Statistical analysis

GraphPad Prism 9.0 was utilized for conducting the statistical analysis, reporting the data as mean ± SD. One-way ANOVA followed by the Bonferroni post hoc test was used for comparing means among groups. *p* ≤ 0.05 indicated statistical significance.

## Results

### Network pharmacology analysis

#### Identification of active components and target genes

**Fig. 2:**
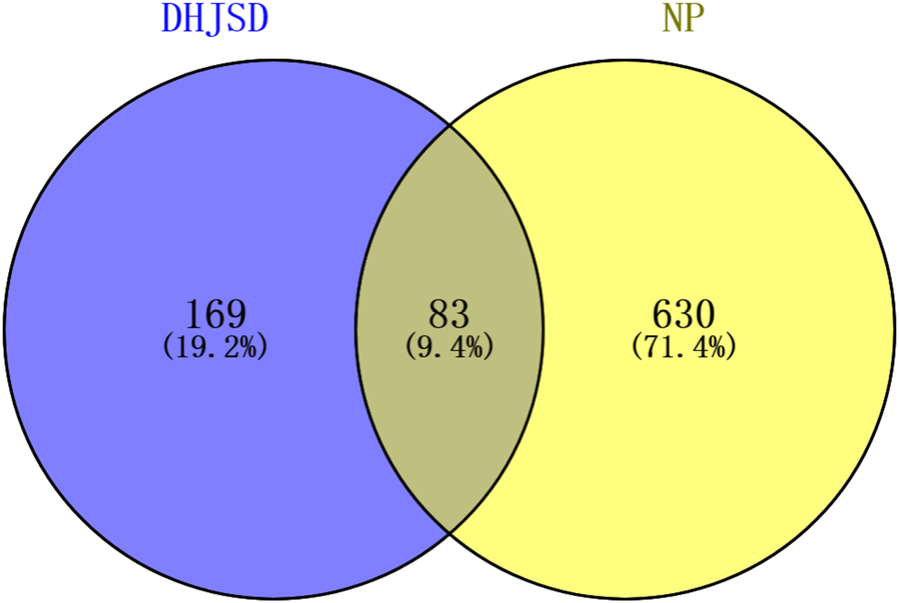
Venn diagram of DHJSD potential targets and NP genes

We identified 180 active components (Additional file 1: Table S1), and 252 potential DHJSD targets were the TCMSP. In addition, 711 and 714 target genes were identified using GeneCards and DisGeNET, respectively (relevance score range: 0.602–113.104, median: 3.418). Of these, 83 target genes were common to the two datasets and were used for further analysis (Fig. 2) (Additional file 1: Table S2).

#### Construction of PPI network

**Fig. 3:**
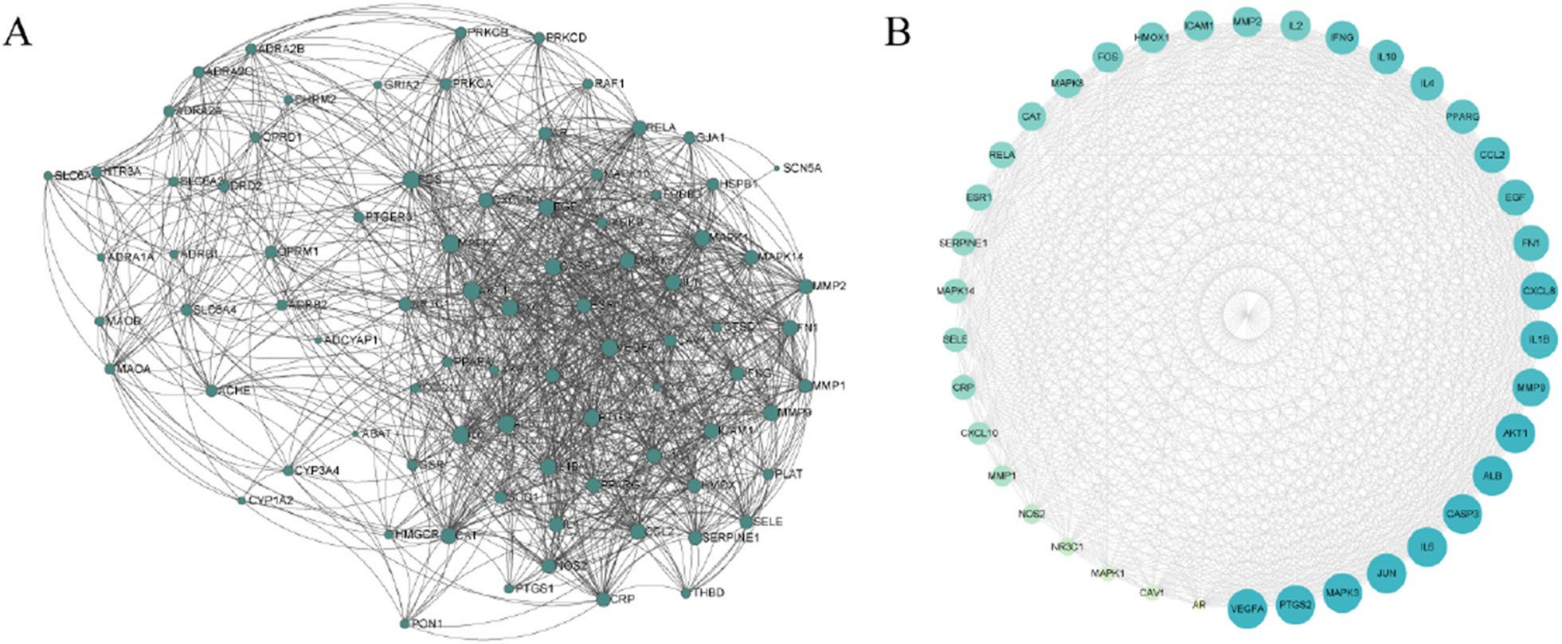
Protein–protein interaction network of DHJSD and NP common targets: **A** the PPI network of DHJSD and NP common targets. **B** The PPI network generated in this study includes 37 nodes larger than the average degree (> 28.361). Among all the core targets, the darker the blue, the larger the circle, the more important it was

The PPI network of the 83 target genes revealed 1177 interactions between the targets (Fig. 3A). The average number of nodes was 28.361, with 37 nodes having a higher degree than the average (Fig. 3B) (Additional file 1: Table S3).

#### GO enrichment analysis

**Fig. 4:**
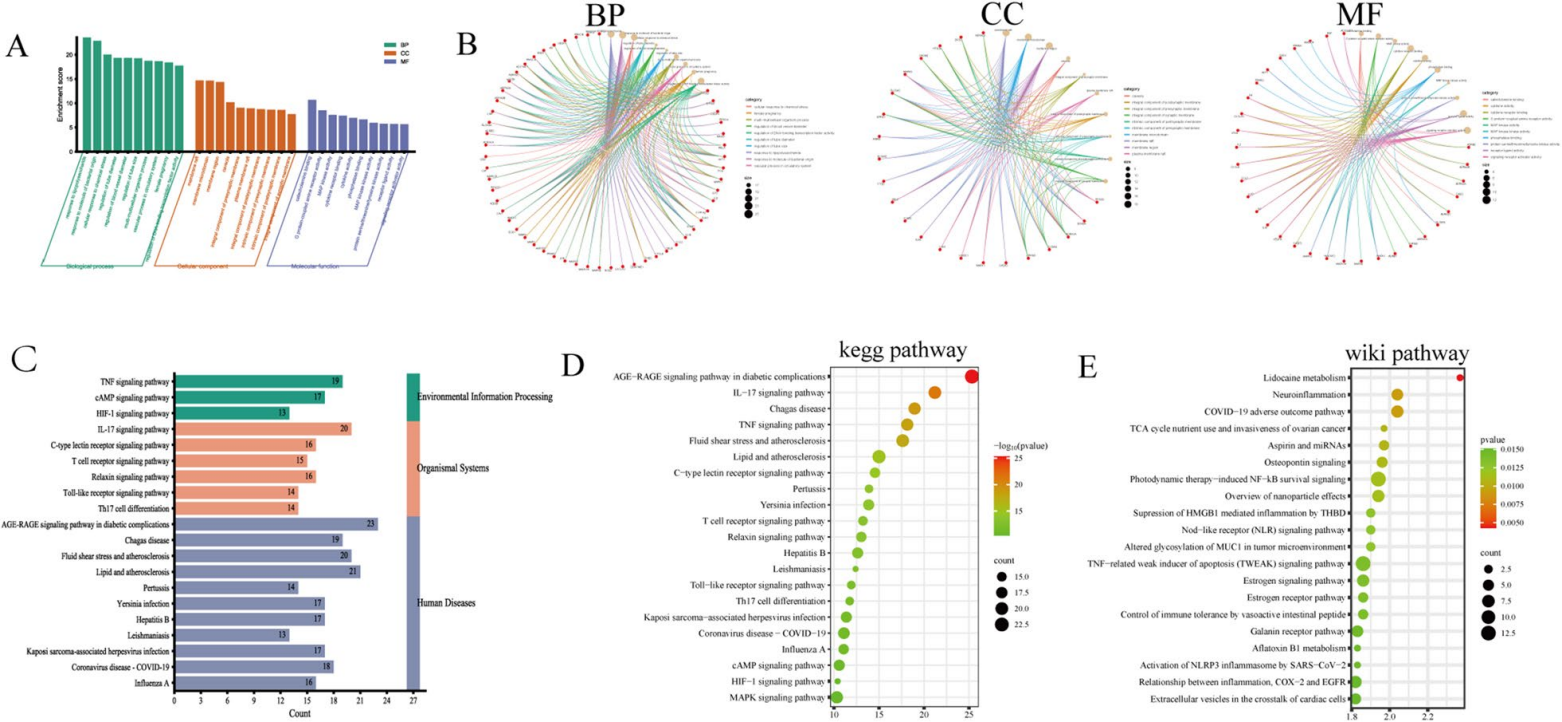
Results of gene ontology and Kyoto encyclopedia of genes and genomes (KEGG) enrichment analysis. **A**, **C** The histogram chart of GO and KEGG enrichment analysis of treating NP targets. **B** The potential key pathways and therapeutic targets for DHJSD treatment of NP. **D** Top 21 of KEGG pathway. **E** Top 20 of WIKI pathway

Based on the GO enrichment analysis, 83 target genes were significantly enriched in 1,525 biological processes (BP), 96 molecular functions (MF), and 55 cell components (CC) (*p* < 0.01) (Additional file 1: Table S4). The top ten terms in each category are shown in Fig. 4A. The potential key pathways and therapeutic targets are shown in Fig. 4B. The top five terms in MFs were catecholamine binding (GO:1901338), G protein-coupled amine receptor activity (GO:0008227), MAP kinase activity (GO:0004707), cytokine receptor binding (GO:0005126), cytokine activity (GO:0005125); in CCs were membrane raft (GO:0045121), membrane microdomain (GO:0098857), membrane region (GO:0098589), caveola (GO:0005901), and presynaptic membrane integral component (GO:0099056); and in BPs were a lipopolysaccharide response (GO:0032496), bacterial origin molecule response (GO:0002237), a circulatory system vascular process (GO:0003018), chemical stress cellular response (GO:0062197), and regulation of tube diameter (GO:0035296).

#### KEGG pathway enrichment analysis

**Fig. 5:**
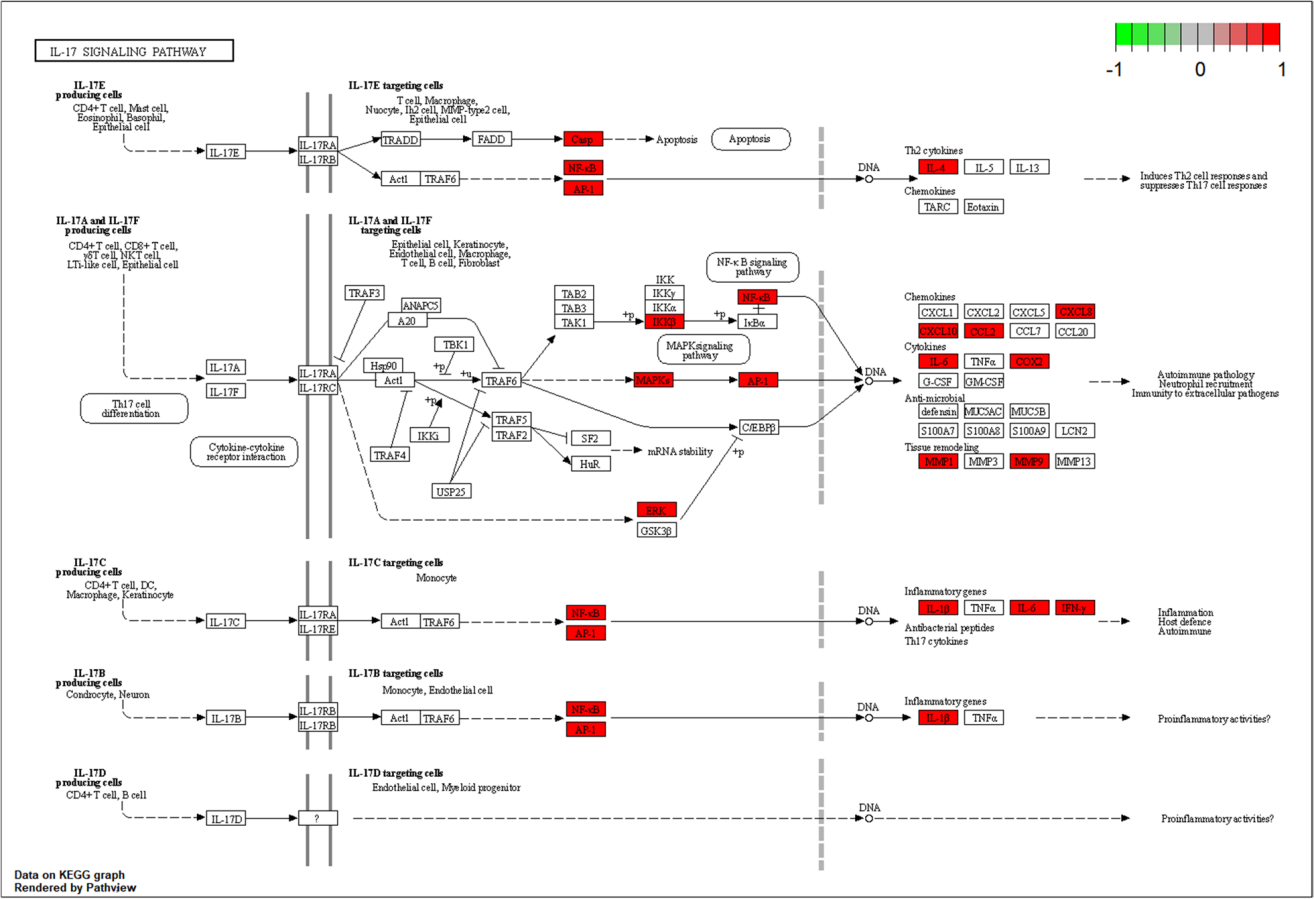
IL-17 signaling pathway (red represents up-regulated genes)

Based on KEGG analysis (Additional file 1: Table S5), 83 target genes were found to be enriched in 163 pathways. Figure 4C, D shows the top 20 KEGG signaling pathways: AGE-RAGE signaling pathway in diabetic complications, IL-17 signaling pathway, Chagas disease, tumor necrosis factor (TNF) signaling pathway, fluid shear stress and atherosclerosis, lipid and atherosclerosis, C-type lectin receptor signaling pathway, Pertussis, Yersinia infection, T-cell receptor signaling pathway, and IL-17 signaling pathway were key pathways (Fig. 5). According to the WIKI database, neuroinflammation was an important physiological process (Fig. 4E).

#### Construction of the compound-target pathway network

**Fig. 6:**
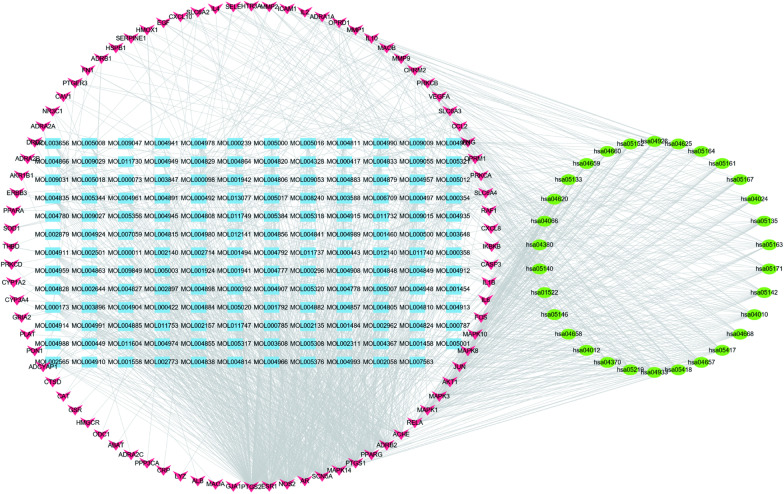
Compound-target-pathway network (blue represents active compounds, red represents common targets, and green represents pathways)

Using 155 active components, 83 target genes, and the top 30 pathways, a compound-target pathway network comprising 268 nodes and 1462 edges was constructed with an average of 10.910 neighbors and a path length of 2.608 (Fig. 6) (Additional file 1: Table S6), revealing the multi-component, -target, and -pathway potential action in neuropathic pain (NP).

#### Molecular docking

**Fig. 7:**
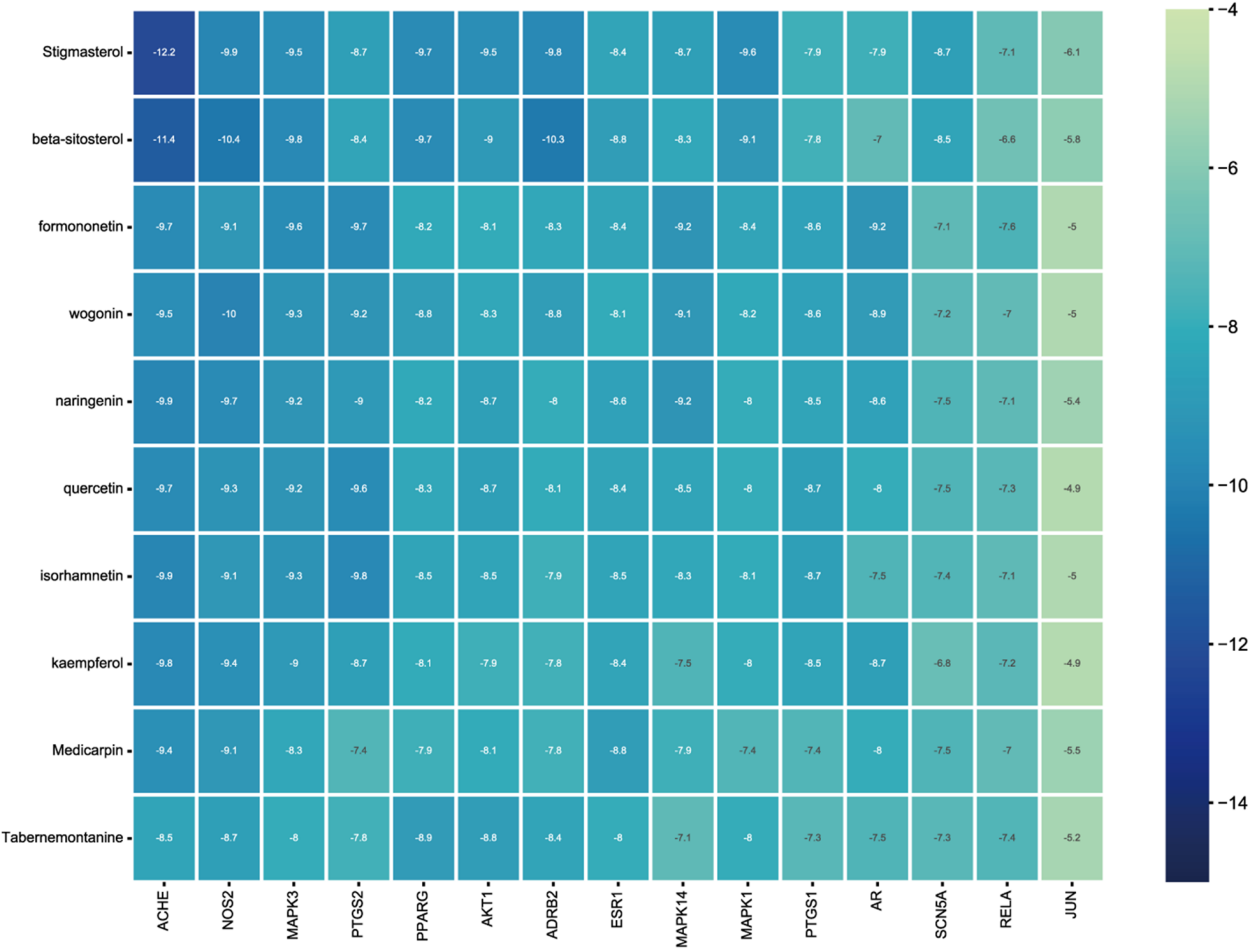
Heat maps of the docking scores

**Fig. 8:**
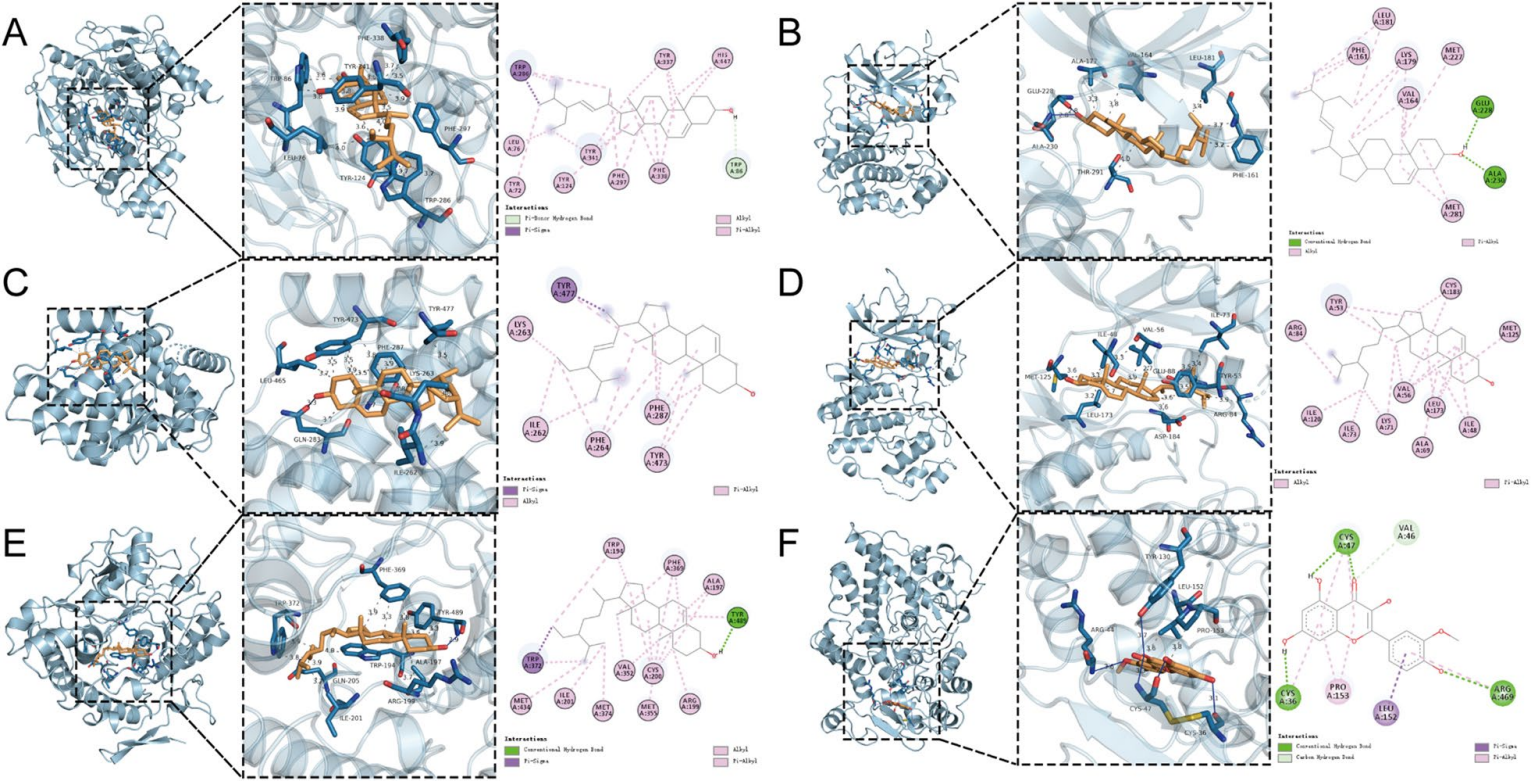
Display of docking results between active components and target genes. **A** ACHE/Stigmasterol; **B** AKT1/Stigmasterol; **C** PPARG/Stigmasterol; **D** MAPK3/beta-sitosterol; **E** NOS2/beta-sitosterol; **F** PTGS2/ Isorhamnetin

The top 10 active components and top 15 target genes in the compound-target pathway network were screened and sorted based on docking scores (Fig. 7) (Additional file 1: Table S7). The six genes with the lowest scores and their corresponding active compounds are shown in Fig. 8. Molecular docking studies indicated that hydrophobic interactions mainly maintain them. Stigmasterol interacted with PHE-338, PHE-297, TYR-341, TYR-124, TRP-86, TRP-286, and LEU-76 on the ACHE protein by hydrophobic interactions. Stigmasterol interacts with GLU-228 and ALA-230 on the AKT1 protein by hydrogen bonding and with ALA-177, VAL-164, THR-291, LEU-181, and PHE-161 via hydrophobic interactions. In PPARG/Stigmasterol, Stigmasterol interacts with GLN-283 on PPARG via hydrogen bonding and with ILE-262, PHE-287, TYR-473, TYR-477, and LYS-263 via hydrophobic interactions. beta-sitosterol hydrophobic interaction with ILE-48, ILE-73, TYR-53, VAL-56, ARG-84. Beta-sitosterol interacts with TRP-194, ALA-197, ARG-199, ILE-201, and GLN-205 on NOS2 via hydrophobic interactions and with TYR-489 via hydrogen bonding. Isorhamnetin formed hydrogen bonds with CYS-36, AGR-44, CYS-47, and TYR-130 on PTGS2, and LEU-152 and PRO-153 formed hydrophobic interactions.

### Experimental validation

#### DHJSD improves cci-induced mechanical allodynia

CCI was performed in rats to evaluate the therapeutic effects of DHJSD on NP. Based on behavioral analysis, the CCI group had significantly lower WMT values on days 1, 4, 7, 10 and 14 postoperation than the sham group (*p* < 0.01). In contrast, the CCI + DHJSD group had significantly higher MWT values on days 4, 7, 10 and 14 postoperation than the CCI group (*p* < 0.05) (Fig. 10C), indicating that DHJSD treatment alleviated mechanical allodynia in CCI rats.

#### Core target verification

**Fig. 9:**
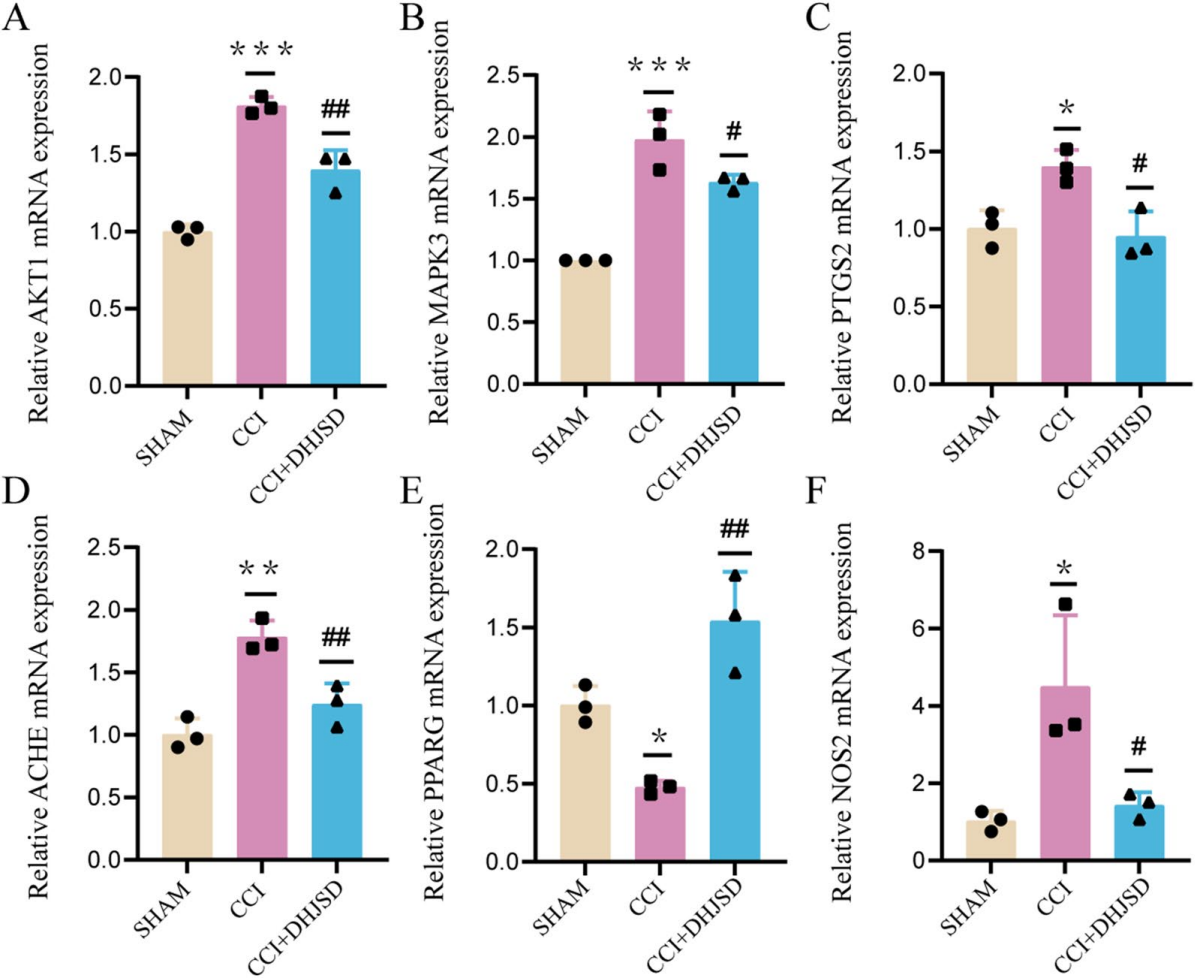
Core target validation. **A**–**F** RT-qPCR analysis of ACHE, NOS2, MAPK3, PTGS2, AKT, and PPARG expression in ipsilateral spinal dorsal horns. (*p =  < 0.05, **p =  < 0.01, ***p =  < 0.001 compared to the sham group, ^#^*p* =  < 0.05, and ^##^*p* =  < 0.01 compared to the CCI group.)

Molecular docking results showed that ACHE, NOS2, MAPK3, PTGS2, AKT1, and PPARG might be key genes associated with DHJSD analgesic effect. RT-qPCR, all ACHE, NOS2, MAPK3, PTGS2, and AKT1 transcriptional levels showed significantly elevated in the CCI group compared to the sham group (*p* < 0.05) and significantly decreased in the CCI + DHJSD group compared to the CCI group (*p* < 0.05). Nevertheless, PPARG had a more significant elevation in the CCI + DHJSD group than in the CCI group (*p* < 0.05) (Fig. 9).

#### DHJSD ameliorated inflammatory response in CCI rats

**Fig. 10:**
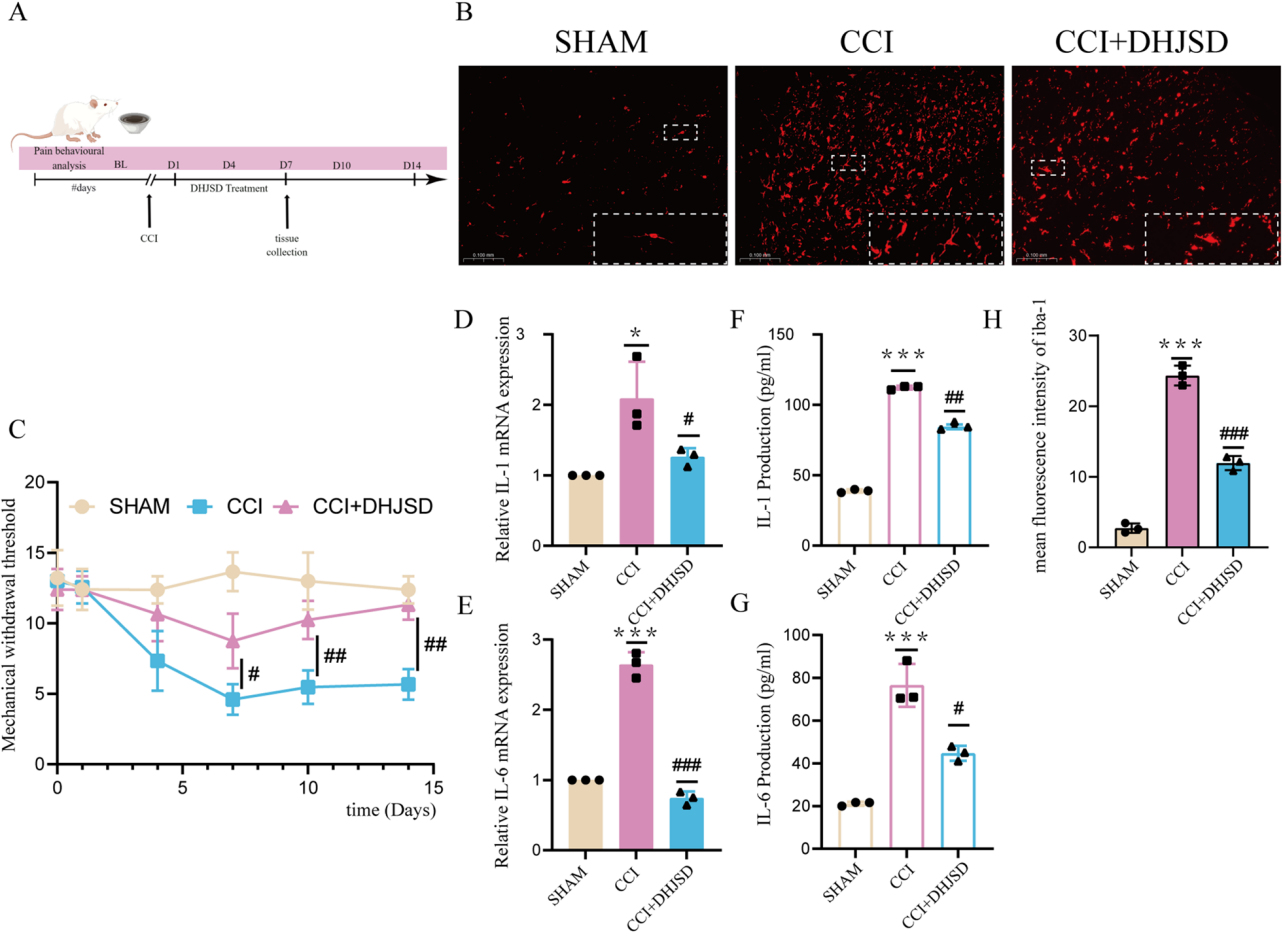
Effect of DHJSD on microglial activation and inflammation in the L4-L6 spinal cord at 7 days post-CCI. After CCI, rats were treated with DHJSD (10.3 mg/kg body weight) for 7 days. In spinal dorsal horns, Iba-1 levels were assessed by immunofluorescence. **A** The timeline of Experiment **B** Iba-1 labeled microglia (red) in spinal dorsal horn. Scale bars, 50 µm. **C** Effects of DHJSD on CCI-induced mechanical allodynia **D**, **E** RT-qPCR analysis of IL-1β and IL-6 expression in the ipsilateral spinal dorsal horns. **F**, **G** ELISA analysis of IL-1β and IL-6 expression in the ipsilateral spinal dorsal horns. **H** quantification of mean fluorescence intensity (*p =  < 0.05, **p =  < 0.01, ***p =  < 0.001 compared to the sham group, ^#^*p* =  < 0.05 and ^##^*p* =  < 0.01 compared to CCI group)

**Fig. 11:**
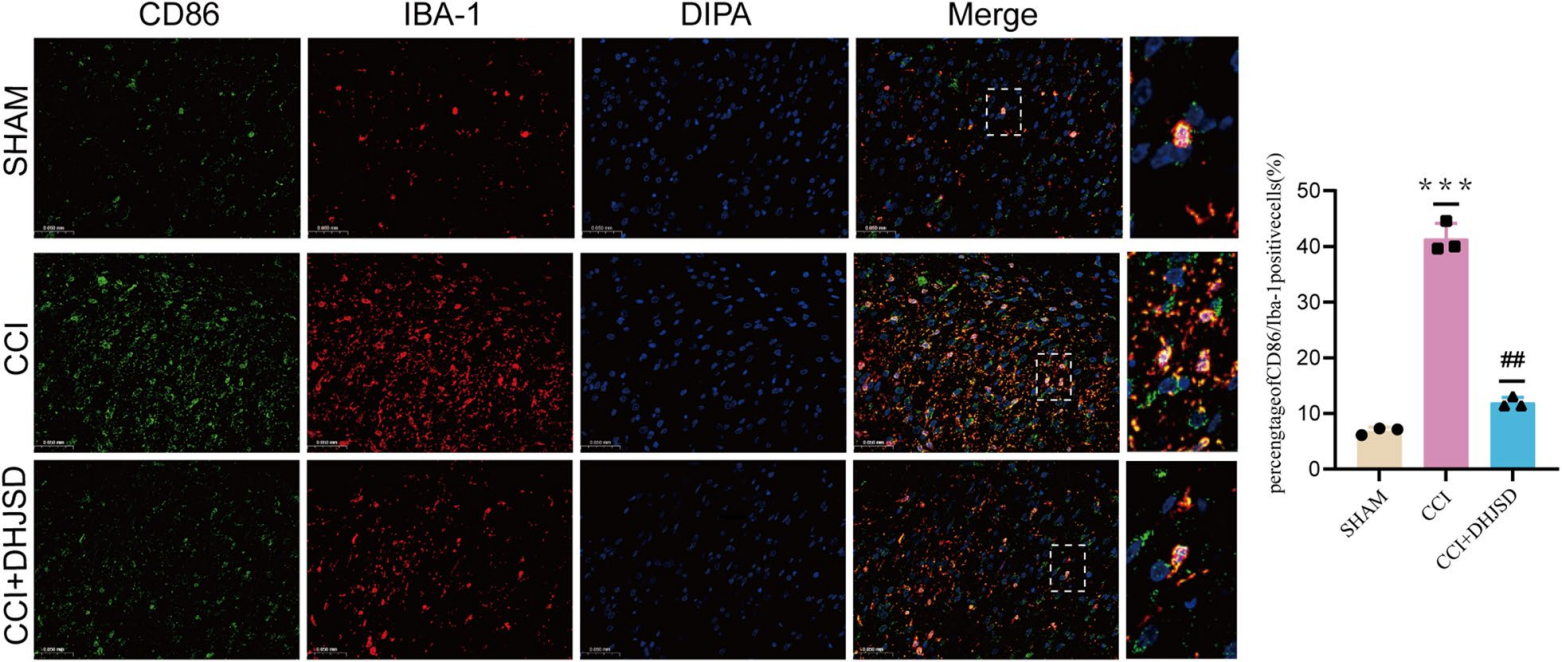
Effect of DHJSD on polarization of microglia in the L4-L6 spinal cord at 7 days post-CCI. In spinal dorsal horns, Iba-1 and CD86 levels were assessed by immunofluorescence. Iba-1 labeled microglia (red) and CD86 labeled *M*1 (green), quantitative analysis of the positive cell percentage in the spinal cord dorsal horn. Scale bar, 50 μm. (***p =  < 0.001 compared to the sham group, ^#^*p* =  < 0.05 and ^##^*p* =  < 0.01 compared to CCI group)

**Fig. 12:**
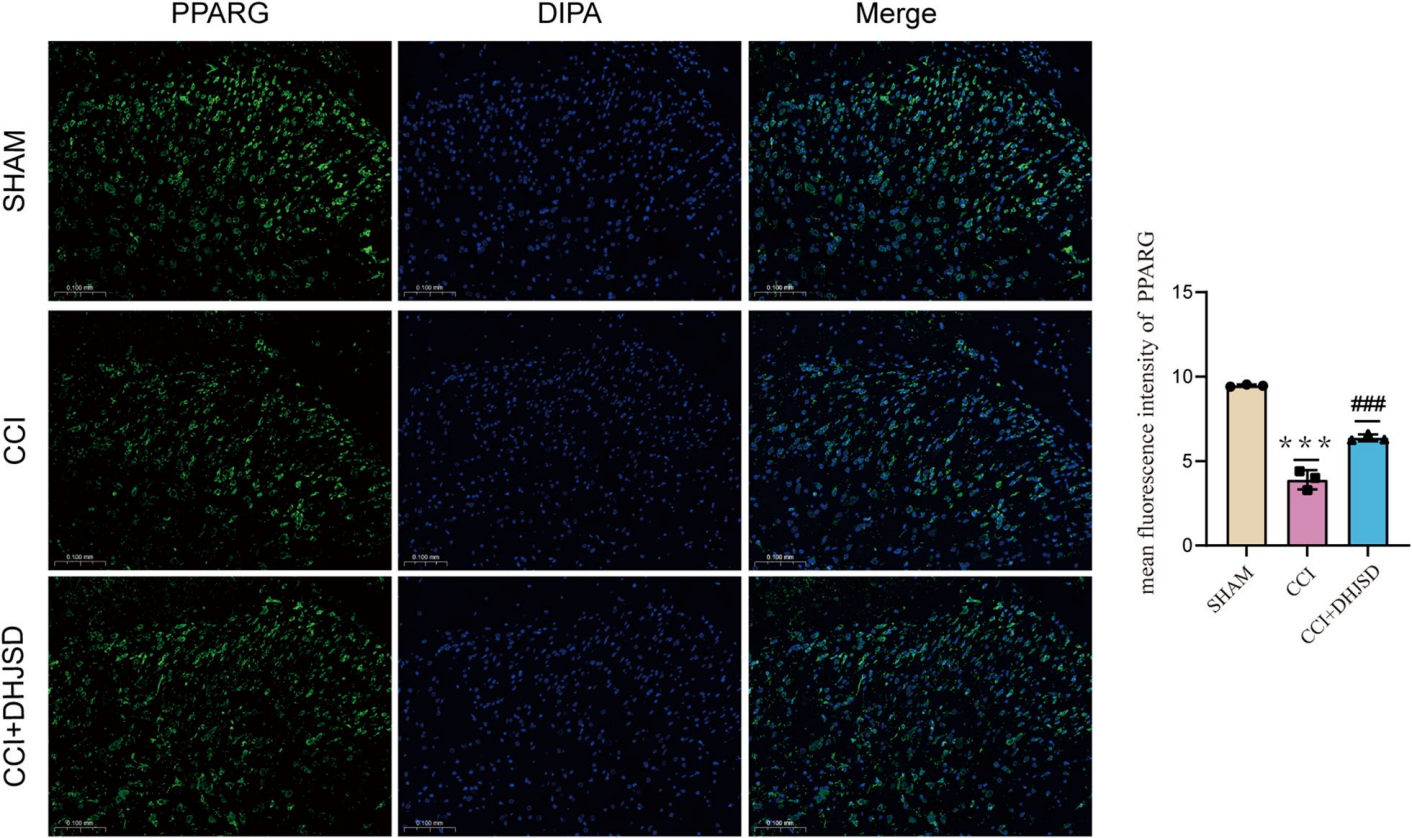
Effects of DHJSD on PPARG levels in L4-L6 spinal cords at 7 days post-CCI. PPARG levels in spinal dorsal horns were assessed by immunofluorescence. PPARG labeled green. Scale bar, 50 µm (***p =  < 0.001 compared to the sham group, ^###^*p* =  < 0.01 compared to CCI group)

Based on network pharmacology, DHJSD treatment of NP was proposed to achieve its therapeutic effects by modulating neuroinflammation, revealing that the inflammatory factors IL-1 and IL-6 were significantly elevated in the CCI group compared to the sham group (*p* < 0.05) and significantly decreased in the CCI + DHJSD group compared to the CCI group (*p* < 0.05) (Fig. 10D, E, F, G). Immunofluorescence microscopy revealed that both Iba-1 and CD86 were significantly overexpressed in the CCI group compared with the sham group (*p* < 0.05). However, CD86 upregulation was reversed in the CCI + DHJSD group (Figs. 10B, 11). On the contrary, PPARG had a more significant elevation in the CCI + DHJSD group than in the CCI group (Fig. 12). Overall, these results suggest that DHJSD can reduce microglial activation and that the inhibition of microglial *M*1 polarization may mediate the relief of inflammation by DHJSD (Additional file 2: Fig S1), PPARG may have been involved in the process.

## Discussion

Neuropathic pain (NP) refers to pain directly caused by an injury or disease involving the somatosensory system [2]; its treatment remains controversial. The etiology of NP is diverse, its pathogenesis is complex, and it is associated with neuroinflammation following nerve injury and may lead to NP [17]. Inflammatory mediators are released by activated microglia, creating a closed-loop system that continually exacerbates damage to peripheral neuronal cells [18, 19]. In addition, microglial activation alone can cause pain hypersensitivity even without neuronal damage or other cellular injuries [20, 21]. Therefore, the modulation of microglial activation via pharmacological approaches is a promising research direction.

TCM is a holistic and systemic approach to treating complex health conditions [22]. DHJSD is a well-known treatment for chronic neuralgia. In the Qianjin Yaofang of the Tang Dynasty, Sun Simiao mentions its usefulness for “dispelling wind and dampness, relieving arthralgia, strengthening the liver and kidney, and replenishing blood and qi” [23]. However, there is a lack of systematic studies on DHJSD for NP; therefore, we aimed to explore the potential analgesic mechanisms of DHJSD by identifying and analyzing the active components, target genes, and related signaling pathways and validating the results in a CCI rat model.

Network pharmacology is a useful tool for revealing the mechanisms of action of TCMs in disease treatment. In our study, 83 target genes identified through multiple database searches were subjected to GO and KEGG analyses, revealing that DHJSD might affect NP by governing numerous BP, CC, and MF besides signaling pathways, and wiki pathways, suggesting the importance of neuroinflammation. Furthermore, DHJSD may synergistically regulate key target genes in the interleukin (IL)-17, tumor necrosis factor (TNF), cAMP, T-cell receptor, Toll-like receptor, HIF-1, and MAPK signaling pathways, also involving response to lipopolysaccharide, cellular response to chemical stress, catecholamine binding, and MAP kinase activity. Previous studies found that IL-17A leads to NF-κB, MAPK, and C/EBP cascades activation, inducing IL-6, IL-1β, and TNFα proinflammatory effects following binding to the corresponding target cell receptors (macrophages, T cells, and epithelial cells) [24–26]. Moreover, MAPK signaling pathway activation is involved in NP by inhibiting ERK1/2, p38, and JNK1/2 activation and decreasing the expression of inflammatory mediators [27, 28]. Wogonin inhibits TLR4-MyD88-TAK1-mediated NF-κB, and MAPK signaling pathways and consequently attenuates iNOS and COX-2 expression, inhibiting neuroinflammation in dorsal root ganglion neurons and improving NP [29]. In conclusion, based on our findings and those of previous studies, we propose that DHJSD treatment of NP may exert its therapeutic effects by modulating neuroinflammation. In our study, we successfully established a CCI rat model and examined spinal cord inflammation levels, revealing that DHJSD improved neuroinflammation by decreasing the expression of inflammatory factors in the spinal cord, in line with previous reports on the anti-inflammatory effects of DHJSD.

The PPI and compound-target pathway networks allowed us to identify the top 10 active components and 15 target genes subjected to molecular docking. The results showed that five ingredients, stigmasterol, beta-sitosterol, formononetin, naringenin, and quercetin, were predicted to be functional ingredients of DHJSD in the treatment of NP with ACHE, NOS2, AKT1, MAPK3, PTGS2, and PPARG.

NOS2 is one of the three nitric oxide synthase (NOS) isoforms, and its end product modulates NP [30]. Both 5-fluoro-2-oxindole and balneotherapy reduce NO production by inhibiting the upregulation of NOS2, which acts as an analgesic [31, 32]. Importantly, iNOS is a marker of *M*1 polarization in microglia that is upregulated in injured neural macrophages and Schwann cells along with other pain mediators (i.e., COX2) [33, 34]. PPAR is a nuclear hormone receptor superfamily ligand-activated transcription factor with 3 isoforms: PPAR-α, PPAR-δ, and PPARG [35]. PPARG is an endogenous anti-inflammatory factor that inhibits the intra-inflammatory response mainly through NF-κB signaling pathway competitive inhibition; thus, it can be used to reduce NP by regulating macrophage infiltration and pro-inflammatory molecule production at the inflammation site [36]. PPARG has also been reported to reverse neuroinflammation by increasing anti-inflammatory factor levels and decreasing spinal pro-inflammatory factor levels [37]. In addition, its overexpression inhibited oligosaccharide-induced CX3CR1 expression and *M*1-type activation in BV-2 microglia, exerting a protective effect against NP [38]. Moreover, isorhamnetin, a quercetin derivative, regulates *M*1/*M2* macrophage polarization and enhances functional recovery in spinal cord injury rats [39]. In conclusion, these findings indicate that the inhibition of microglial *M*1 polarization may mediate the anti-inflammatory effects of DHJSD.

Microglia are the immune cells of the CNS. Neuroinflammation caused by microglia is also involved in NP development [40]. When external stimulus signals are received, microglia undergo two polarization states: pro-inflammatory (*m*1) and anti-inflammatory (*m2*) [41]. In the early stages of neuroinflammation, *m*1-like microglia secrete TNF, IL-1, and IL-6 as pro-inflammatory substances, which damage neural networks [42–44]. Notably, suppression of *M1* polarization in microglia attenuates NP [37, 45].

In our study, ACHE, MAPK3, and PTGS2 were significantly downregulated, whereas PPARG was upregulated in the CCI + DHJSD group compared to the CCI group. Moreover, DHJSD significantly suppressed NOS2 expression in the spinal cord and decreased CD86/Iba-1 positive cell number in the dorsal horn. These results showed that DHJSD markedly improved neuroinflammation, partly by reducing microglial *M1* polarization in the L4–6 spinal cord and the expression of inflammatory factors.

But there are limitations to our experiments, we focused only on changes in microglia. Recent studies have found that astrocyte-microglia interactions are particularly important for the treatment of neuropathic pain, and bidirectional communication between astrocytes and microglia can modulate central nervous system responses through the secretion of multiple cytokines and inflammatory mediators [46]. Astrocytes are abundant in the central nervous system (CNS). Although glial cells were initially thought to provide trophic support for neurons only, it is now evident that astrocytes are important for developing and maintaining neuropathic pain. Astrocytes appear to proliferate after nerve injury, and the intrathecal injection of reagents that inhibit the proliferation of astrocytes in nerve-injured rats results in the restoration of tactile tenderness [47]. The modulation of *M1*-type microglial polarization can similarly affect astrocytes. Reactive astrocytes exhibit heterogeneous and multifaceted functions in SCI, such as providing trophic and metabolic support to neurons, inhibiting axonal growth, and modulating inflammation [48, 49]. Necrotic astrocytes provide limited support for neuronal survival, and astrocyte necrosis inhibition can rescue the reactive astrocyte neurotrophic function of glial cells. Researchers have found that inhibition of *M1* microglia may induce astrocyte necrosis by activating TLR4/MyD88 signaling, thereby favoring neuronal cell survival [50]. MpHE can regulate microglial phagocytic activity and prevent the pro-inflammatory *M1* phenotype through PPAR-r/CD36-dependent mechanisms, inhibiting astrocyte proliferation and neuroinflammation amelioration [51]. Thus, we speculate that DHJSD may inhibit *M1*-type microglial polarization, thereby reducing astrocyte proliferation and treating neuropathic pain.

In conclusion, using network pharmacology, we systematically analyzed the analgesic mechanism of DHJSD and found that stigmasterol, beta-sitosterol, formononetin, naringenin, and quercetin were the active chemical components responsible for its action, while ACHE, NOS2, AKT1, MAPK3, PTGS2, and PPARG were the core targets. The results of the in vivo experiments also suggested that DHJSD significantly ameliorated neuroinflammation following NP treatment, in part by decreasing *M1* polarization and inflammatory factor expression in the microglia. In future studies, we will focus on exploring the specific therapeutic mechanisms of DHJSD in NP, thereby contributing to developing new analgesic drugs for the effective treatment of NP with mild or no side effects.

## Conclusions

Network pharmacology, molecular docking, and animal experiments were combined to study the analgesic mechanisms of DHJSD. Based on our data, DHJSD may regulate microglial polarization in the spinal cord to alleviate neuroinflammation; however, further research is required to validate our results. Overall, exploring the active components of DHJSD and the regulatory mechanisms underlying microglial cell activation in the rat spinal cord may help develop new analgesic drugs to effectively treat NP.

### Supplementary Information

**Additional file 1. Table S1**: Chemical compounds of 15 herbs in DHJSD. **Table S2**: Targets of DHJSD and NPs. **Table S3**: PPI analysis. **Table S4**. GO enrichment analysis data. **Table S5**. KEGG pathway enrichment analysis data. **Table S6.** C-T-P analysis data. **Table S7**: Molecular docking data.

**Additional file 2.** **Fig S1:** M2 polarisation.

## Data Availability

The data used to support the findings of this study are available from the corresponding author upon request.

## Abbreviations

DHJSD: Duhuo Jisheng decoction
NP: Neuropathic pain
TCM: Traditional Chinese medicine
PPI: Protein–protein interaction
GO: Gene ontology
KEGG: Kyoto encyclopedia of genes and genomes
CCI: Chronic constriction injury
MWT: Mechanical withdrawal threshold
GABA: Gamma-aminobutyric acid
APR: Angelicae Pubescentis Radix
IL-6: Interleukin 6
IL-1β: Interleukin 1 beta
TCMSP: Traditional Chinese Medicine Systems Pharmacology
PDB: Protein data bank
PBS: Phosphate-buffered saline
BP: Biological processes
CC: Cell components
MF: Molecular functions
