# Supplementary figures and images for: Duhuo Jisheng decoction alleviates neuroinflammation and neuropathic pain by suppressing microglial M1 polarization: a network pharmacology research
Source: J Orthop Surg Res. 2023 Aug 28;18:629. doi: 10.1186/s13018-023-04121-9 (PMC10463324; doi:10.1186/s13018-023-04121-9)

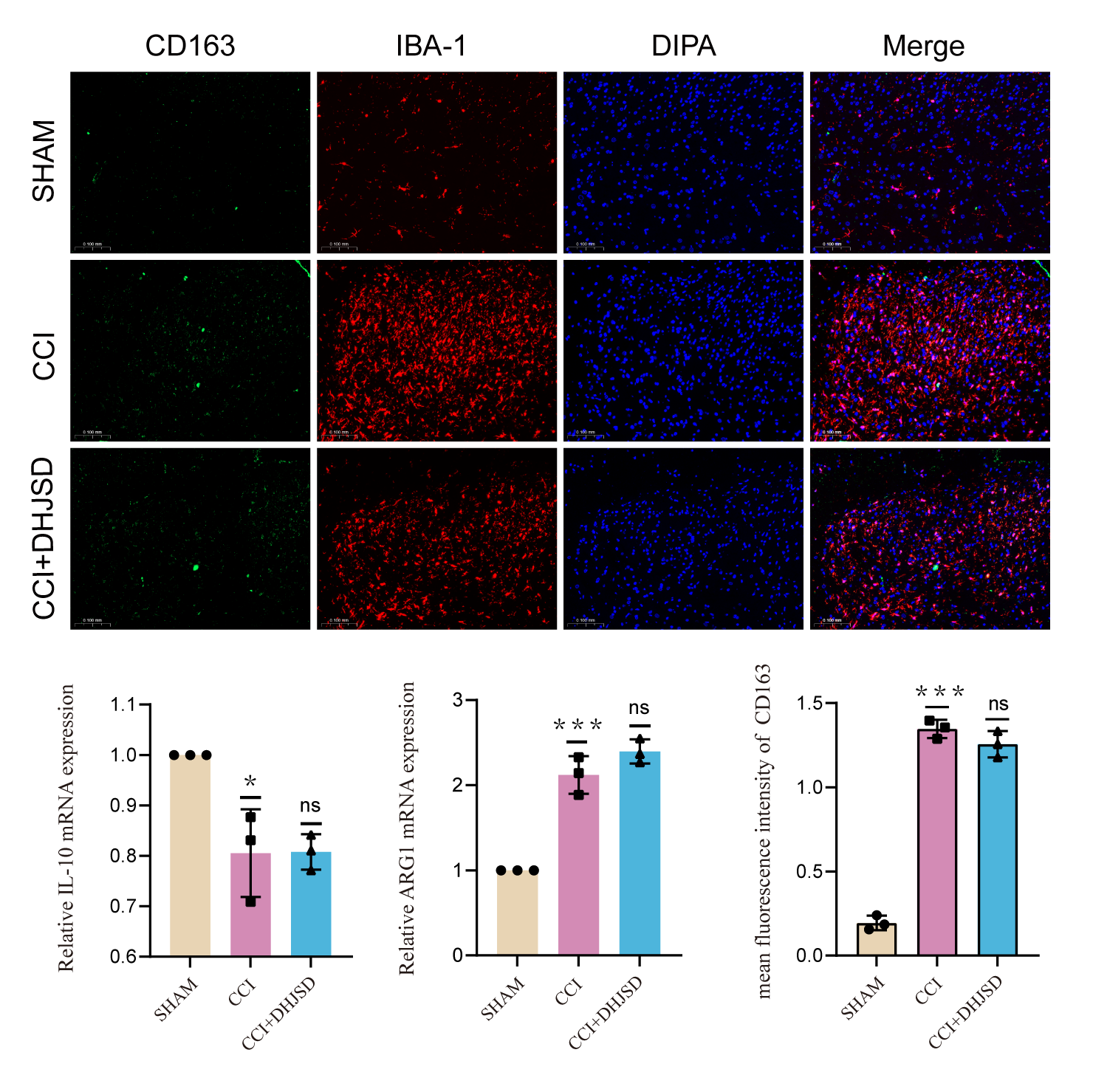

Supplement: Supplementary file 2 — Additional file 2. Fig S1: M2 polarisation. [file 13018_2023_4121_MOESM2_ESM.tif]
